# Transcriptional profiling of innate immune responses in sheep PBMCs induced by *Haemonchus contortus* soluble extracts

**DOI:** 10.1186/s13071-019-3441-8

**Published:** 2019-04-25

**Authors:** Si Wang, Dandan Hu, Chaoyue Wang, Xinming Tang, Mengze Du, Xiaolong Gu, Jingxia Suo, Min Hu, Rui Fang, Xingquan Zhu, Xichen Zhang, Aifang Du, Xun Suo, Xianyong Liu

**Affiliations:** 1 State Key Laboratory of Agrobiotechnology, Key Laboratory of Zoonosis of Ministry of Agriculture, National Animal Protozoa Laboratory and College of Veterinary Medicine, China Agricultural University, Beijing, China; 2 State Key Laboratory of Agricultural Microbiology, College of Veterinary Medicine, Huazhong Agricultural University, Wuhan, Hubei China; 3 State Key Laboratory of Veterinary Etiological Biology, Key Laboratory of Veterinary Parasitology of Gansu Province, Lanzhou Veterinary Research Institute, Chinese Academy of Agricultural Sciences, Lanzhou, Gansu China; 4 College of Veterinary Medicine, Jilin University, Changchun, Jilin China; 5 Institute of Preventive Veterinary Medicine & Zhejiang Provincial Key Laboratory of Preventive Veterinary Medicine, Zhejiang University, Hangzhou, Zhejiang China

**Keywords:** *Haemonchus contortus*, C-type lectin receptors (CLRs), NOD-like receptor (NLR), Hydroxycarboxylic acid receptor 2 (HCAR2), Transcription factors

## Abstract

**Background:**

Pattern recognition receptors (PRRs) can recognize pathogen-associated molecular patterns and activate downstream signalling pathways, resulting in modulation of host immunity against pathogens. Here, we investigated whether PRR-mediated recognition is involved in host immune responses to the blood-feeding nematode *Haemonchus contortus*.

**Methods:**

During blood-feeding, *H. contortus* secretes immune-modulating antigens into host blood. Therefore, we stimulated sheep peripheral blood mononuclear cells (PBMCs) with *H. contortus* soluble extract (HcAg) and performed transcriptional profiling.

**Results:**

HcAg upregulated two genetically linked CLRs (CLEC2L and KLRG2), two NLRs attenuating inflammation (NLRP12 and NLRC3) and one G protein-coupled receptor with potent anti-inflammatory effects (HCAR2). Furthermore, several Th2-related transcription factors (ATF3, IRF4, BCL3 and NFATC) were also upregulated, which may confer anti-inflammatory type 2 immune responses to HcAg.

**Conclusions:**

Together, our preliminary studies provide new insights into how the host innate immune system controls type 2 immunity to *H. contortus*. Further work will be needed to identify *H. contortus* products recognized by the host innate immune system and determine the Th2 polarization ability of these putative PRR ligands.

**Electronic supplementary material:**

The online version of this article (10.1186/s13071-019-3441-8) contains supplementary material, which is available to authorized users.

## Background

Control of type 1 immunity against microbes by the innate immune system has been extensively studied. A set of host germline-encoded receptors, also known as pattern recognition receptors (PRRs), can recognize pathogen-associated molecular patterns (PAMPs), which are conserved molecules derived from microbes [1]. The recognition of PAMPs by PRRs activates downstream signalling pathways, inducing the transcription of pro-inflammatory cytokines [2]. In contrast, parasitic worms have developed intricate strategies to shift host immune responses towards an anti-inflammatory type 2 immunity [3–5], and little is known about how the innate immune system controls type 2 immunity.

Several immunomodulators derived from helminth products have been shown to be recognized by host PRRs. The excretory and secretory product ES-62 from the rodent filarial nematode *Acanthocheilonema viteae* is recognized by TLR4, biasing the host immune response towards an anti-inflammatory/Th2 phenotype [6]. LNFPIII, a carbohydrate immunomodulator derived from soluble extracts of *Schistosoma mansoni* eggs (SEA) drives Th2 polarization *via* activation of the atypical NF-kB family member BCL3 in a DC-SIGN- and TLR4-dependent manner [7, 8]. Furthermore, lyso-phosphatidylserine (PS), a lipid derived from *S. mansoni* eggs and adult worms, activates TLR2 and polarizes the maturation of dendritic cells, resulting in Th2 skewing [9, 10].

*Haemonchus contortus*, due to its blood-feeding behaviour and the potential for rapid development of large burdens to the host, is recognized as one of the most pathogenic nematodes in small ruminants [11]. Consistent with other helminth infections, *H. contortus* also polarizes host immune responses towards an anti-inflammatory/Th2 phenotype [12]. However, little is known about the mechanism of Th2 polarization and whether PRR-mediated recognition is involved.

Here, we tried to dissect host innate immune responses to *H. contortus* infection by RNA-seq. After *H. contortus* soluble extract (HcAg) stimulation, sheep PBMCs upregulated several germline-encoded receptors and Th2-promoting transcription factors, repressing the transcription of the pro-inflammatory cytokine IL-12. Impaired IL-12 signalling resulted in Th2 commitment, shifting host immune responses towards an anti-inflammatory type 2 immunity. Our work suggests how the host innate immune system regulates type 2 immune responses to *H. contortus* infection.

## Methods

### Animals

Local female crossbred sheep (3 to 6 months old) were housed indoors at China Agricultural University (CAU). We fed these sheep with hay and whole shelled corn, and daily health observations were performed throughout the experiment. Faecal parasitic egg counts (FECs) were monitored for at least one week before starting the experiment. Three sheep with no helminth eggs were used for blood collection and transcriptional profiling.

### Cells

Peripheral blood mononuclear cells (PBMCs) were isolated from sheep peripheral blood by the standard Ficoll gradient centrifugation method and resuspended in RPMI 1640 medium (Macgene, Beijing, China) supplemented with 10% FBS, 50 U/ml penicillin, 50 μg/ml streptomycin, 2 mM l-glutamine and 50 μM 2-mercaptoethanol. PBMCs were seeded in 60 mm flat-bottom tissue culture dishes (Corning, New York, USA) (3 × 10^7^ cells/dish) and incubated at 37 °C with 5% CO_2_ for 1.5 h. Non-adherent cells were removed by washing twice with phosphate-buffered saline (PBS). Adherent cells were cultured as above and rested overnight before stimulation with HcAg.

### Preparation of *H. contortus* antigen (HcAg)

*Haemonchus contortus* soluble extracts used as HcAg were obtained as described previously with minor modifications. Briefly, adult worms were obtained from a *H. contortus*-infected sheep abomasum, washed several times in PBS containing 50 U/ml penicillin and 50 μg/ml streptomycin, and then homogenized and centrifuged. The supernatant was collected after filtration (0.22 μm) and stored at −20 °C for subsequent use. Protein concentrations were determined by the bicinchoninic acid assay (BCA).

To determine the effects of carbohydrates in HcAg, periodate treatment was carried out as described by Velupillai et al. [13]. Briefly, HcAg was initially dialyzed against 0.05 M acetate buffer (pH 4.5) before addition of 100 mM sodium meta-periodate (Sigma-Aldrich, Shanghai, China). The reaction vial was then gently mixed for 1 h in the dark and halted with sodium borohydride (Sigma-Aldrich) at a final concentration of 50 mM for 30 min at room temperature. Finally, HcAg was dialyzed against PBS, and protein concentrations were measured by BCA.

To determine the effects of proteins in HcAg, HcAg (50 μg/ml) was treated with 50 μg/ml Proteinase K (Sigma-Aldrich, Shanghai, China) for 1 h, and the enzyme activity was heat inactivated.

To eliminate the effects of endotoxin contamination, HcAg (50 μg/ml) was mixed with polymyxin B (PMB, 50 μg/ml) (InvivoGen, San Diego, USA), which is a cyclic cationic polypeptide antibiotic that blocks the biological effects of LPS through binding to lipid A.

### *In vitro* stimulation of sheep PBMCs

Sheep PBMCs were left unstimulated or stimulated for 6 h with native HcAg (Native, 50 μg/ml), proteinase K-treated HcAg (PK, 50 μg/ml), periodate-treated HcAg (PI, 50 μg/ml), polymyxin B-treated HcAg (PMB, 50 μg/ml) or LPS (10 ng/ml; InvivoGen).

### RNA-seq

Total RNA was isolated from stimulated or unstimulated cells and subjected to RNA sequencing on an Illumina HiSeq instrument (Beijing, China). Sequenced reads (40–50 million per sample) were aligned to the *Ovis aries* genome using the Tophat 2 program, and differences in gene expression between samples were analysed using the DEGseq R package (v.1.20.0) [14]. The *P*-values were adjusted using the Benjamini & Hochberg method. Corrected *P*-values of 0.005 and log2 (fold change) of 1 were set as the threshold for significantly differential expression. Gene ontology (GO) enrichment analysis of differentially expressed genes was implemented by the GOseq R package, in which gene length bias was corrected. GO terms with a corrected *P*-value less than 0.05 were considered significantly enriched by differentially expressed genes. As the GO-term ‘response to molecule of bacterial origin’ was enriched in transcripts responding to each stimulation, we analysed the transcripts belonging to this GO term and filtered them out to eliminate the effects of bacterial stimulation.

Datasets for the reads are available from the NCBI Sequence Read Archive (SRA) submission (accession number SRP132649).

### Quantitative real-time PCR (qPCR)

RNA was extracted with chloroform, and RNA concentration was determined using a DeNovix spectrophotometer DS 11 (Wilmington, USA). Reverse transcription of total RNA was performed using the PrimeScript RT Kit with gDNA Eraser according to the protocol provided by the manufacturer (TaKaRa, Beijing, China). Gene expression was analysed on an ABI 7500 Real-Time PCR system (Applied Biosystems, Foster City, USA) using the SYBR Green method (TaKaRa). Data were normalized to GAPDH, and the comparative ΔCT method (2^−ΔΔCt^) was applied to determine relative quantification of gene expression (shown as fold change). Primers were designed using NCBI’s Primer-blast (details are shown in Additional file 1: Table S1).

### Statistical analysis

Results are shown as the mean ± SEM. Statistical analysis was performed with GraphPad Prism software v.7.0 (GraphPad Software). qPCR data was analysed by the ordinary one-way ANOVA or two-way ANOVA. The results were considered significant at *P < 0.05, **P < 0.01, ***P < 0.001; ns, not significant.

## Results

### Transcriptional profiling revealed that *H. contortus* soluble extracts modulated the immune responses of sheep PBMCs

**Fig. 1:**
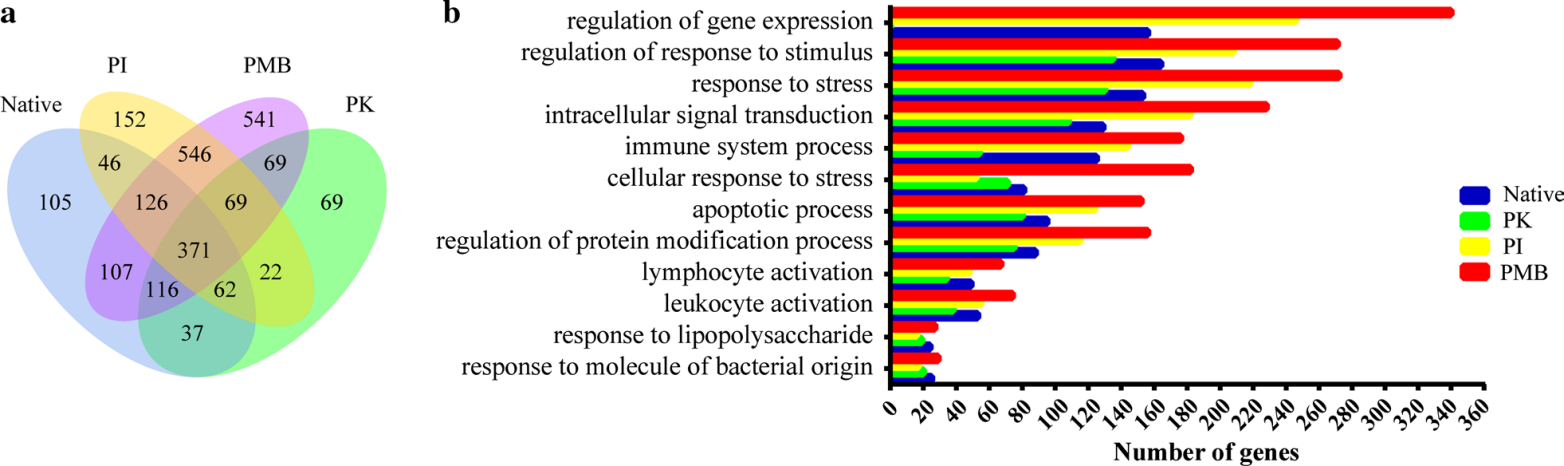
Transcriptional profiling of sheep PBMCs stimulated by *H. contortus* soluble extract (HcAg). Sheep PBMCs were stimulated for 6 h with native HcAg (Native, 50 μg/ml), proteinase K-treated HcAg (PK, 50 μg/ml), periodate-treated HcAg (PI, 50 μg/ml) or polymyxin B-treated HcAg (PMB, 50 μg/ml), and transcriptional profiling was determined by RNA-seq. **a** Venn diagram showing overlap between differentially expressed genes of sheep PBMCs after stimulation. **b** Gene ontology-term analysis of differentially expressed genes. *P*_corrected_ < 0.001

To investigate whether PRR-mediated recognition regulates the immune responses of sheep to its blood-feeding nematode *H. contortus*, we stimulated sheep PBMCs for 6 h with *H. contortus* soluble extracts (HcAg) and probed gene expression of the stimulated PBMCs by RNA sequencing. Differential expression analysis revealed that HcAg modulated a substantial number of genes’ expression (Fig. 1a). Gene ontology-term analysis of differentially expressed genes showed that HcAg modulated the host’s immune system processes (Fig. 1b). Proteinase K or periodate treatment reduced the immune-modulating capacity of *H. contortus* soluble extracts, indicating the necessity of proteins and carbohydrates in the extracts.

### *Haemonchus contortus* soluble extracts upregulated several germline-encoded receptors of sheep PBMCs

**Fig. 2:**
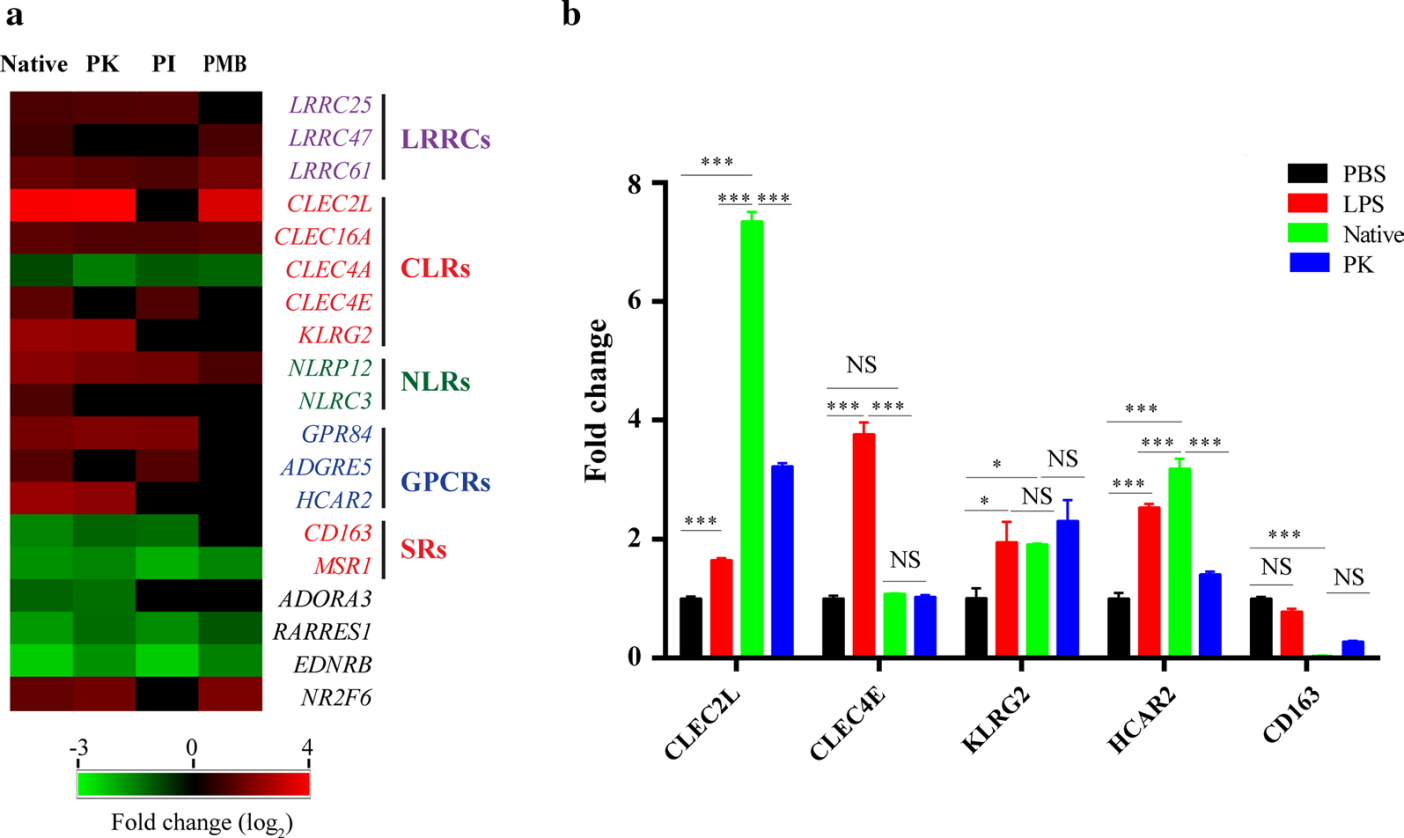
Detailed analysis and validation of differentially expressed germline-encoded receptors of sheep PBMCs after stimulation. **a** Heatmap showing differentially expressed transcripts related to LRRCs, CLRs, NLRs and GPCRs after native, proteinase K-treated (PK), periodate-treated (PI) or polymyxin B (PMB)-treated HcAg stimulation. **b** Quantitative RT-PCR (qPCR) analysis of selected genes in sheep PBMCs. Error bars show SEM (*n* = 3). qPCR data was analysed by ordinary one-way ANOVA. ***P < 0.0001, **P < 0.01, *P < 0.05. *Abbreviation*: ns, not significant

To investigate whether *H. contortus* soluble extracts can modulate PRR expression in sheep PBMCs, we analysed the transcripts related to germline-encoded receptors. Upon HcAg stimulation, sheep PBMCs up- or downregulated several germline-encoded receptors, including C-type lectin receptors (CLRs), NOD-like receptors (NLRs), G protein-coupled receptors (GPCRs), scavenger receptors (SRs) and some undefined leucine-rich repeat containing proteins (LRRCs) (Fig. 2a). Quantitative real-time PCR (qPCR) validated the expression pattern of most germline-encoded receptors (Fig. 2b). C-type lectin domain family 2 member L (CLEC2L) was the most significantly increased transcript (ANOVA: *F*_(3,8)_ = 3188, *P* < 0.0001), followed by HCAR2 (hydroxycarboxylic acid receptor 2) (ANOVA: *F*_(3,8)_ = 268.8, *P* < 0.0001) and KLRG2 (killer cell lectin-like receptor 2) (ANOVA: *F*_(3,8)_ = 8.2, *P *= 0.0211). Furthermore, HcAg significantly downregulated the macrophage scavenger receptor CD163 (cluster of differentiation 163) (ANOVA: *F*_(3,8)_ = 731.1, *P* < 0.0001). The activation or repression of these germline-encoded receptors may motivate specific signalling pathways, conferring the anti-inflammatory/Th2 immune responses to *H. contortus*.

### *Haemonchus contortus* soluble extracts upregulated several PRRs downstream signalling molecules in sheep PBMCs

**Fig. 3:**
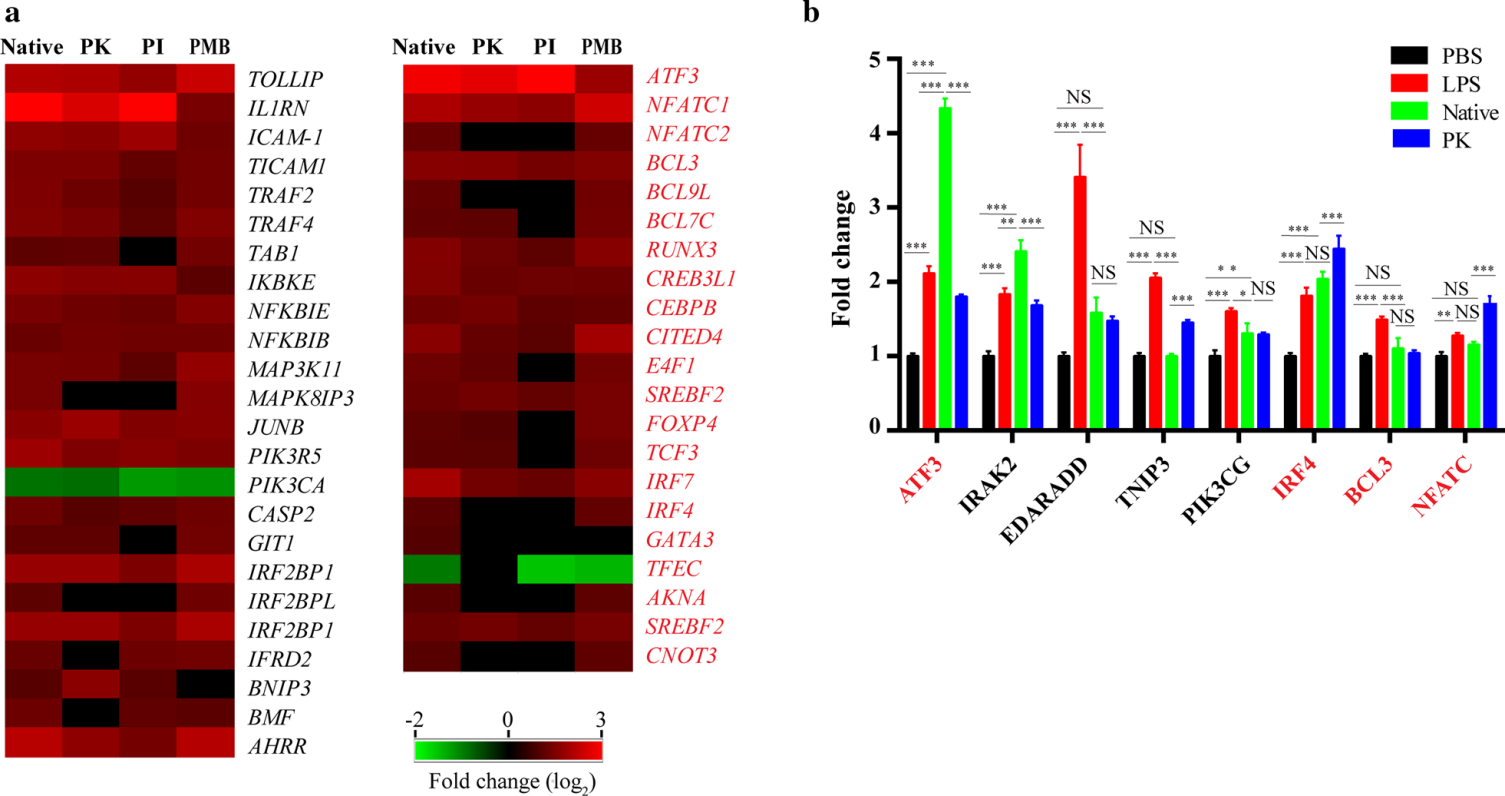
Detailed analysis and validation of differentially expressed signalling pathways of sheep PBMCs after stimulation. **a** Heatmap showing differentially expressed transcripts related to signalling cascades and transcription factors. **b** Validation of selected genes by qPCR. Error bars show SEM (*n* = 3). qPCR data was analysed by ordinary one-way ANOVA. ***P < 0.0001, **P < 0.01, *P < 0.05. *Abbreviation*: ns, not significant

We next sought to investigate whether *H. contortus* soluble extracts can modulate the downstream signalling molecules of PRRs. Upon HcAg stimulation, sheep PBMCs upregulated the necessary signal transduction molecules involved in PRRs signalling (Fig. 3a). Furthermore, HcAg upregulated negative regulators of inflammation (TOLLIP, ATF3 and BCL3), and transcription factors involved in Th2 skewing (IRF4, NFATC1 and NFATC2) (Fig. 3a, b). Notably, activating transcription factor 3 (ATF3) was the most significantly increased transcript among all the transcripts involved in signal transduction (ANOVA: *F*_(3,8)_ = 897.8, *P *< 0.0001). Thus, HcAg modulated the expression of PRRs downstream signalling molecules to suppress inflammation and promote Th2 polarization.

### *Haemonchus contortus* soluble extracts induced PRRs downstream effector molecules’ expression in sheep PBMCs

**Fig. 4:**
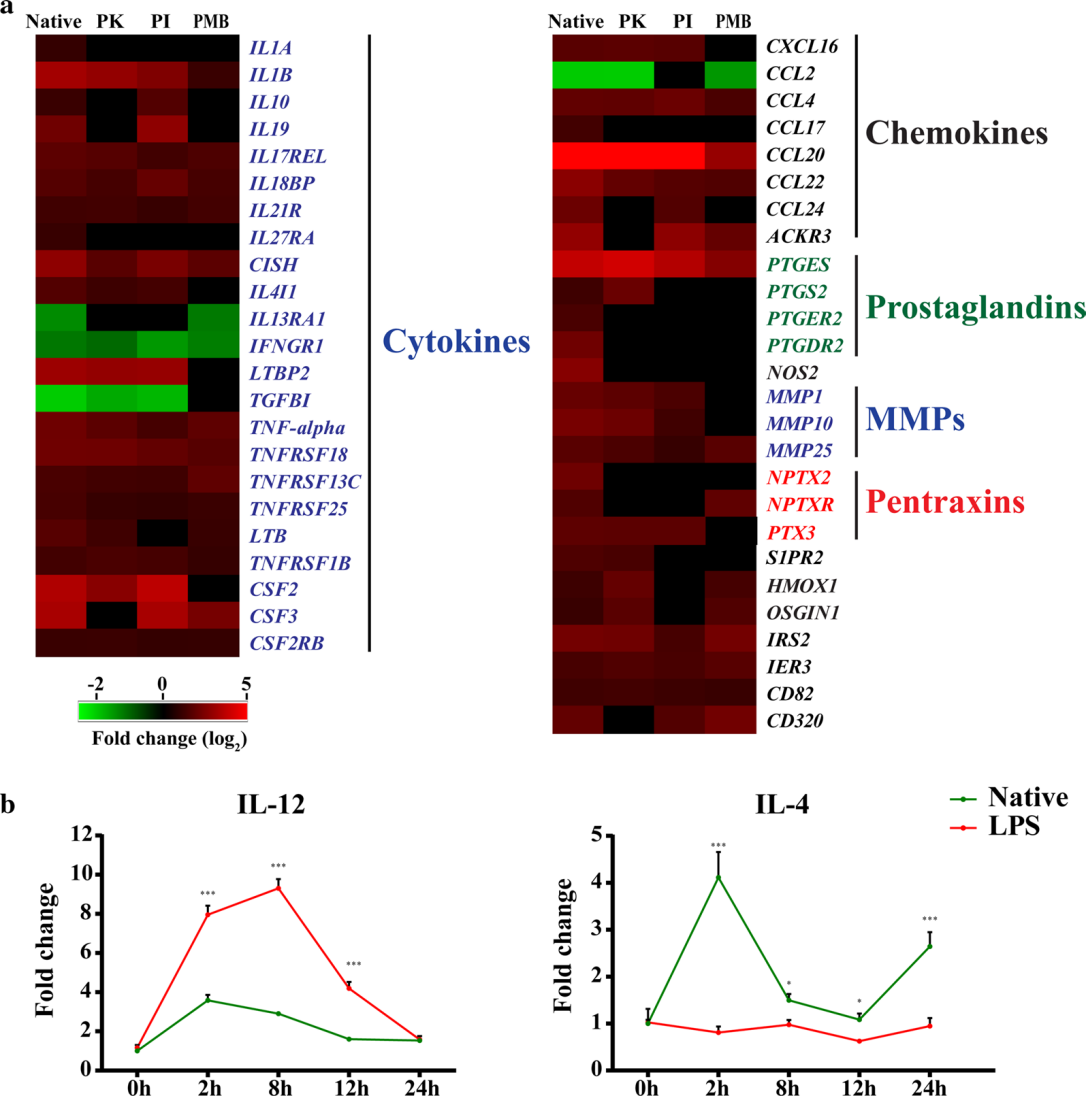
Detailed analysis and validation of differentially expressed effector molecules of sheep PBMCs after stimulation. **a** Heatmap showing differentially expressed transcripts related to signalling cascades and transcription factors. **b** Validation of selected genes by qPCR. Error bars show SEM (*n* = 3). qPCR data was analysed by two-way ANOVA. ***P < 0.0001, **P < 0.01, *P < 0.05. *Abbreviation*: ns, not significant

Given that HcAg modulated the expression of PRRs and downstream signalling molecules, we then analysed the effector molecules induced by HcAg. In addition to differentially expressed cytokines and chemokines, HcAg also promoted the expression of effector molecules related to host immune responses to helminth infection, including prostaglandins, MMPs and NOS (Fig. 4a). The upregulation of IL4I1 (interleukin 4 induced 1) and downregulation of IFNGR1 (interferon gamma receptor 1) indicated Th2 skewing after HcAg stimulation (Fig. 4a). We further confirmed this Th2 polarization by detecting the expression level of IL-12 and IL-4 of sheep PBMCs after stimulation (Fig. 4b). HcAg induced IL-4 expression significantly, with a peak at 2 h after stimulation (ANOVA: *F*_(1,19)_ = 174, *P* < 0.0001). In contrast, the Th1 stimulus LPS tended to induce a high level of IL-12 (ANOVA: *F*_(1,4)_ = 580.7, *P* < 0.0001). IL-4 is a strong Th2 polarization signal, whereas IL-12 is a strong Th1 polarization signal, and impaired IL-12 signal would promote Th2 commitment [15, 16]. Thus, increased expression of IL-4 and decreased expression of IL-12 may contribute to HcAg-induced Th2 skewing.

## Discussion

**Fig. 5:**
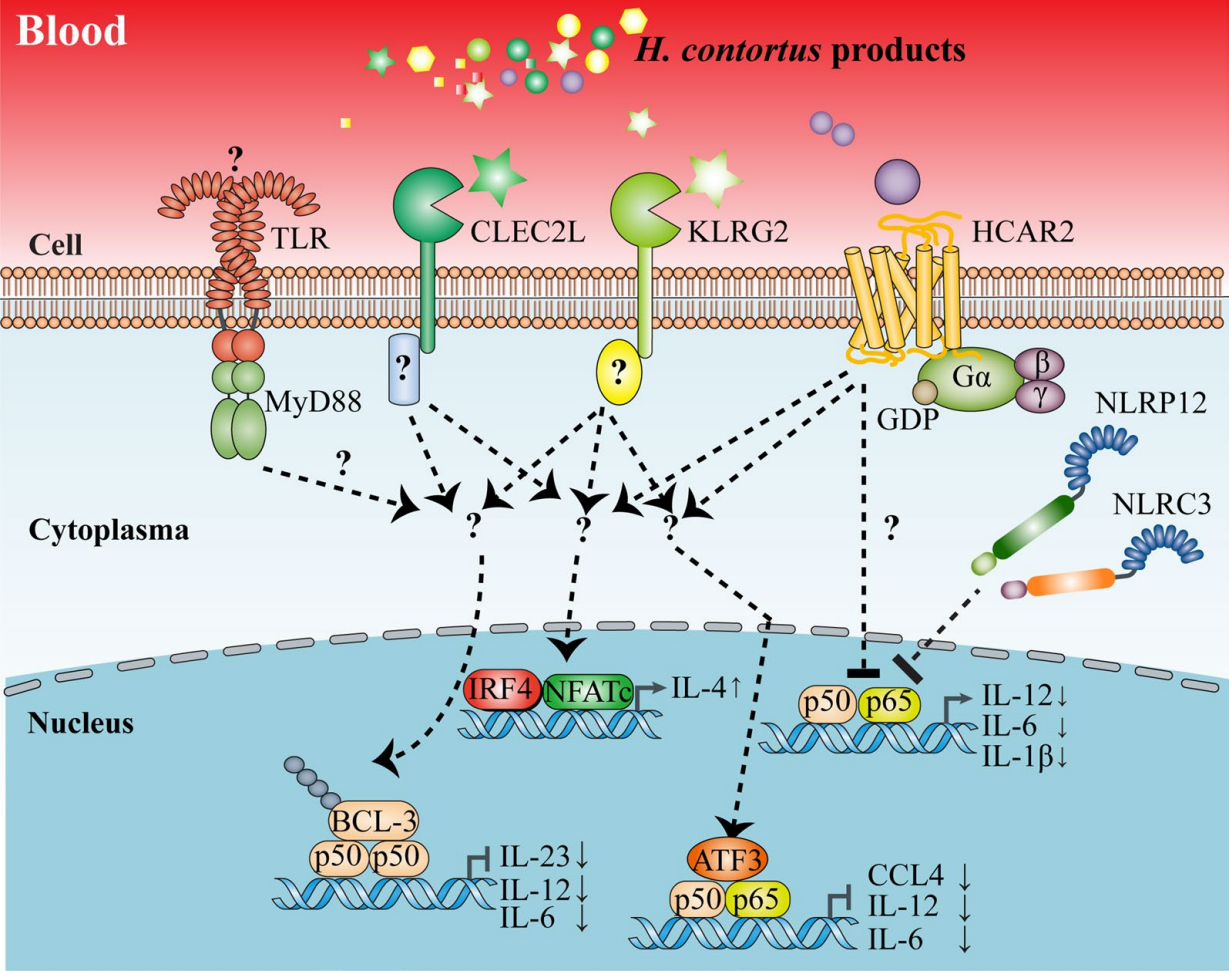
Postulated mechanisms of how the innate immune system controls type 2 immunity to *H. contortus*. *Haemonchus contortus* immunomodulators, when being secreted into blood during feeding, upregulate germline-encoded receptors (CLEC2L, KLRG2, NLRP12, NLRC3 and HCAR2) in circulating blood cells. These receptors activate downstream signalling molecules (ATF3, IRF4, BCL3 or NFATC) and subsequently repress the transcription of pro-inflammatory cytokines (IL-12). Loss of IL-12 signalling results in Th2 commitment, shifting host immune responses towards an anti-inflammatory type 2 immunity

Our study revealed how host innate immune system respond to HcAg stimulation. Several germline-encoded receptors (CLEC2L, KLRG2, NLRP12, NLRC3 and HCAR2) and downstream signalling molecules (ATF3, IRF4, BCL3 and NFATC1) were upregulated, resulting in anti-inflammatory responses and Th2 skewing (Fig. 5).

Several CLRs have been shown to recognize helminth infection and inhibit NF-κB-mediated pro-inflammatory gene expression by activating specific transcriptional factors [8, 17–21]. Our study showed that HcAg stimulation significantly upregulated the expression of two genetically linked CLRs (CLEC2L and KLRG2) in sheep PBMCs. However, little is known about their function [15]. We are particularly interested in identifying the function of CLEC2L and KLRG2 in recognizing *H. contortus* infection.

Both NLRP12 (NLR family, pyrin domain-containing 12) and NLRC3 (NLR family, CARD domain-containing 3) can suppress inflammation by negatively regulating NF-κB signalling [16, 17]. HCAR2, previously termed GPCR109A, mediates profound anti-inflammatory effects in multiple tissues [18, 19]. The endogenous ligand for HCAR2 is beta hydroxybutyrate (β-OHB), a ketone body produced by the liver when an individual is in a negative energy balance [22]. HCAR2 is also a receptor for niacin and butyrate, which are commensal microbiota products [23]. It has been shown that HCAR2 signalling significantly reduced NF-κB activation levels, promoting anti-inflammatory responses [24, 25]. The main consequence of helminth infection is nutritional disturbance or malnutrition [26]. It is yet to be determined whether hydroxybutyrate (produced by the host) or its analogue (produced by the parasite) is increased during *H. contortus* infection, both of which activate HCAR2 and result in anti-inflammatory responses.

HcAg upregulated Th2-related transcription factors ATF3, IRF4, BCL3 and NFATC1. ATF3 is rapidly induced by various stress signals, including nutrient deprivation, oxidative stress and DNA damage [27]. Induced ATF3 serves as a negative regulator of NF-κB-related gene transcription, leading to the decreased production of pro-inflammatory cytokines after TLR activation [28, 29]. BCL3 is an atypical NF-κB family member, which can interfere with p65–p50 (classical NF-κB) binding and repress TLR-induced pro-inflammatory cytokine expression [8]. BCL3 activation during *S*. *mansoni* infection represses IL-12 while enhancing Th2 cell-attracting chemokine expression, shifting Th cell differentiation from Th1 to Th2 polarization [8]. Both IRF4 and NFAT are key regulators of Th2 cell development [30–32]. IRF4 is required for the differentiation of PDL2^+^ DCs and M2 macrophages, which are important in immunity to parasitic helminths [33–35]. Activation of the Ca^2+^-calcineurin-NFAT cascade was also seen during the neuropeptide NMU-mediated ILC2 activation, which promotes the transcription of IL-5, IL-13 and amphiregulin [36]. Upregulation of the above transcription factors in sheep PBMCs by HcAg reveals the intricacy and complexity of the host’s immune network in response to this blood-feeding parasite infection.

*Haemonchus contortus*, the most widespread parasite of ruminants, was previously known to be a good model for studying anthelmintic drug resistance. Here, we tried to dissect host innate immune responses to this blood-feeding parasite. *Haemonchus contortus* has a lancet tooth for piecing blood vessels when feeding. During blood-feeding, the salivary gland of this parasite secretes immune-modulating antigens into the host blood, so we used host PBMCs to perform all of the assays. Indeed, it is better to use *H. contortus* salivary gland soluble extracts to stimulate sheep PBMC and investigate how this parasite modulates host immune responses during blood-feeding. However, it is hard to collect enough parasite salivary glands which is why we chose whole parasite soluble extracts as stimuli.

## Conclusions

Collectively, our preliminary work postulates a new picture of how the host innate immune system integrates control of *H. contortus* infection. *Haemonchus contortus* soluble extracts triggered the upregulation of two genetically linked CLRs (CLEC2L and KLRG2), two NLRs attenuating inflammation (NLRP12 and NLRC3), and one G protein-coupled receptor with potent anti-inflammatory effects (HCAR2). These germline-encoded receptors may activate Th2-related transcription factors (ATF3, IRF4, BCL3 or NFATC1), repressing transcription of the pro-inflammatory cytokine IL-12. Loss of IL-12 signalling results in Th2 commitment, shifting host immune responses towards an anti-inflammatory type 2 immunity. Further work will be needed to identify *H. contortus* products recognized by the host innate immune system and determine the Th2 polarization ability of these putative PRR ligands.

## Additional file

**Additional file 1: Table S1.** Primers used for real-time PCR.

## Abbreviations

PRRs: pattern recognition receptors
PBMCs: peripheral blood mononuclear cells
HcAg: *H. contortus* soluble extract
CLRs: C-type lectin receptors
NLR: NOD-like receptor
GPCRs: G protein-coupled receptors
SRs: scavenger receptors
LRRCs: leucine-rich repeat containing proteins
PMB: polymyxin B
FECs: faecal parasitic egg counts
GO: gene ontology
PBS: phosphate-buffered saline
BCA: bicinchoninic acid assay
SRA: sequence read archive
qPCR: quantitative real-time PCR
CLEC2L: C-type lectin domain family 2 member L
KLRG2: killer cell lectin-like receptor 2
CD163: cluster of differentiation 163
NLRP12: NLR family, pyrin domain-containing 12
HCAR2: hydroxycarboxylic acid receptor 2
NLRC3: NLR family, CARD domain-containing 3
CLEC4A: C-type lectin domain family 4, member A
ATF3: activating transcription factor 3
TOLLIP: Toll interacting protein
IRF4: interferon regulatory factor 4
BCL3: B-cell lymphoma 3-encoded protein
NFATC1: nuclear factor of activated T-cells, cytoplasmic 1
NFATC2: nuclear factor of activated T-cells, cytoplasmic 2
IL4I1: interleukin 4 induced 1
IFNGR1: interferon gamma receptor 1
β-OHB: beta hydroxybutyrate
