# Supplementary material for: Transcriptional profiling of innate immune responses in sheep PBMCs induced by Haemonchus contortus soluble extracts
Source: Parasit Vectors. 2019 Apr 25;12:182. doi: 10.1186/s13071-019-3441-8 (PMC6482558; doi:10.1186/s13071-019-3441-8)
Supplement: Supplementary file 1 — Additional file 1: Table S1. Primers used for real-time PCR. [file 13071_2019_3441_MOESM1_ESM.docx]

**Additional file 1: Table ‎S1. Primers used for real-time PCR.‎**

| **Gene** | **Primer sequence (5'-3')** | **Amplified product (bp)** |
| --- | --- | --- |
| GAPDH | F: CCATGTTTGTGATGGGCGTG | 154 |
|  | R: GCGTGGACAGTGGTCATAAGT |  |
| Actin | F: ACTGGGACGACATGGAGAAGATC | 131 |
|  | R: CTGGGTCATCTTCTCACGGTTG |  |
| CLEC2L | F: AACACCTCCAGCACCGAAGA | 94 |
|  | R: ACAAGAGCGGTCAGATGAGTG |  |
| CLEC4E | F: ACCAGCATCACAAAGCACAG | 160 |
|  | R: TGGCTGGAACTTTTTCTCATCAC |  |
| CD163 | F: TCAGAAGCGAAGACAGAGACA | 146 |
|  | R: CTCTAAGAACCCACAGACCTGA |  |
| KLRG2 | F: AGCCGGAGCCGAGTTAGAAAT | 109 |
|  | R: CAAGCTGTTCTCGGGACTCTCA |  |
| HCAR2 | F: GCAAATGAACAGACACGCCAAG | 83 |
|  | R: GCAGGAAGCAGATGATGAAGACA |  |
| ADORA3 | F: CCTTTGCTGGCTGGTGTCATTC | 100 |
|  | R: GCAGGGTAGGAAGGTGAGGTTT |  |
| LRRC4 | F: ACAACACCTACAAACCAGCACAC | 123 |
|  | R：CCTTGTCCTTGGTATGGGTCTGA |  |
| ATF3 | F: TCGCCATCCAGAACAAGCAC | 101 |
|  | R: CTCGGCTTTGGTGACTGACAT |  |
| TNIP3 | F: AGGATGCCTTGAAACTGGAGTG | 96 |
|  | R: CCATTTCTGTTCGCATCTCCTCA |  |
| PIK3CG | F: AAAGCACAGTGGGCAACACG | 130 |
|  | R: CAGTAGCCAGCACAGGAATAAACG |  |
| IRAK2 | F: ACAGATGACGTGGACAATTCCAG | 108 |
|  | R: CATGCCTTCTTCATCCGCTGT |  |
| EDARADD | F: ACTGCCCAGCAAATTCCGATG | 100 |
|  | R: CAGGACACATCCTTGCTCACATCT |  |
| IRF4 | F: TGTGGGAGAACGAGGAGAAGAG | 188 |
|  | R: CTTGTTCAGAGCACACCGCA |  |
| BCL3 | F: TTCTGTCAGCATCACCACCCTC | 103 |
|  | R: GGAGATGAGGGAGGAAGGAAGAAA |  |
| NFATC1 | F: ACAACTTTCTTCAGGACTCCAAGG | 154 |
|  | R: CTGGTTATCCTCTGGTTGCGGA |  |
| IL-12B | F: CCCACATTCCTACTTCTCCCTGAC | 95 |
|  | R: CTTTGGCTGAGGTTTGGTCTGT |  |
| IL-6 | F: AGAACGAGTTTGAGGGAAATCAGG | 116 |
|  | R: GTGTGTGGCTGGAGTGGTTATT |  |
| CCL20 | F: CCCAGGCTGCTATCAAACATCAC | 166 |
|  | R: GCTTGCTGCTTCTGACTTGCT |  |
| IL-1RN | F: TCTGTCTCCTCCTCTTCCTGTTC | 99 |
|  | R: GGTTGACATCCCAGATCCTGAAG |  |
| IL-1β | F: ACAGGAAATGAGCCGAGAAGTGG | 143 |
|  | R: GCAGGGTCGGTGTATCACCTTT |  |
| IL-10 | F: TGATGCCACAGGCTGAGAAC | 138 |
|  | R: CCACCGCCTTGCTCTTGTTT |  |
| CCL4 | F: CTGCTGCTTCTCTTACACCCTG | 70 |
|  | R: GCTGCTGGTCTCGTAGTAGTCA |  |
| CCL24 | F: CCCTCTTCCTGTTGCATTACCTTC | 84 |
|  | R: GATGCTCCTGTTGGTAAGCTGGTA |  |
| CSF2 | F: TTCTGAACGACAGCACTGACAC | 81 |
|  | R: GCTCCTGGGAGTCAAACATTTCAG |  |
| MMP1 | F: CCGCATCTCCCAAATGGACTTC | 168 |
|  | R: GATGCTCTTCACCGTTCTTGGAAA |  |
| PTGES | F: CTGTTCCTGGGCTTTGTCTACTC | 78 |
|  | R: CAGGAAGAAGACCAGGAAGTGC |  |
